# Orbital fractures treated in a university hospital of southern Italy: epidemiology, outcomes and prognostic factors resulting from 538 retrospectively analyzed cases

**DOI:** 10.1007/s10006-024-01236-z

**Published:** 2024-04-01

**Authors:** Walter Colangeli, Francesco Ferragina, Elvis Kallaverja, Chiara Celano, Maria Giulia Cristofaro

**Affiliations:** 1 Department of Experimental and Clinical Medicine, Unit of Maxillofacial Surgery, “Magna Graecia” University, Viale Europa, 88100 Catanzaro, Italy; 2 “Magna Graecia” University, Viale Europa, 88100 Catanzaro, Italy

**Keywords:** Orbital fractures, Maxillofacial surgery, Epidemiology, Traumatology, Outcomes and prognostic factors

## Abstract

**Purpose:**

Orbital fractures are common injuries and represent an interesting chapter in maxillofacial surgery. This retrospective study analyses data collected from 528 patients surgically treated at the University Hospital “Magna Graecia”, Catanzaro, Italy, from 1st January 2007 to 31st January 2021.

**Methods:**

The inclusion criteria were a diagnosis of orbital bone fracture, complete clinical and radiological records, and a minimum follow-up of 12 months. We analyzed gender, age, etiology, fracture type, treatment, timing of repair, and associated complications.

**Results:**

The most frequent cause of trauma was road accidents (37.88%), followed by domestic accidents (25.95%). The manifestation of diplopia (72.35%), infraorbital nerve hypoesthesia (53.41%), extrinsic eye movement limitation (51.70%), and enophthalmos (41.29%), determined the indication for surgery. Our trauma team preferred the sub-eyelid approach (79.36%). The study shows a statistical significance in the correlation between the severity of the herniation of the lower rectus muscle and the presence of preoperative diplopia (*p*-value = 0.00416); We found the same statistical significance for the post-postoperative diplopia (*p*-value = 0.00385). Patients treated two weeks after the trauma show a higher rate of diplopia and a greater limitation of long-term post-operative eye movements than those treated within two weeks (diplopia 23.08% vs. 15.56%; eye movements limitation 13.33% vs. 7.69%). Early surgical treatment (> 14 days) reduces the likelihood of functional and structural damage to the lower rectus muscle.

**Conclusion:**

Our data will support future maxillofacial traumatology studies, and the education and prevention measures taken will reduce the incidence of orbital trauma.

## Introduction

Orbital fractures (OF) have an incidence of 40–70% of all facial bone fractures and the orbital floor is the most involved as it represents the thinnest wall [1–6]. They are more common in males between 21 and 30 years [2, 7]. The most common etiology is road accidents and accidental falls [7–11]. According to the Cramer et al. classification, we distinguish OF into pure (confined to orbital walls) and isolated orbital fractures (involving adjacent bones) [1–6]. Often associated signs and symptoms are diplopia, eye movement limitation (entrapment of the inferior rectus muscle), hypo-anesthesia (infraorbital nerve involvement), and enophthalmos. The main objective of the surgery is to restore the continuity of the bone walls to reestablish the pre-trauma orbital volume through various reconstructive approaches.

Recent advances in technology have improved the way these traumas are handled. The three-dimensional printing made it possible to create specific implants built to adapt to the anatomy of the patient using pre-operative CT data. In the literature, there are several conflicting studies concerning the management of orbital fractures, especially concerning the factors that influence the outcome of surgery, such as the extent of the fracture and the repair time. This retrospective study retrospectively analyzes the orbital fractures treated surgically at the Department of Maxillofacial Surgery of the University Hospital “Magna Graecia”, one of the largest reference centers for maxillofacial trauma in Southern Italy. It also evaluates epidemiology and focuses on the associations between age, sex, etiology, fracture site, repair time, material, and technique used for reconstruction. The analysis of our data can contribute to future prospective studies on orbit fractures by suggesting prevention measures that could reduce the incidence of this type of trauma and define a management protocol to improve treatment results.

## Materials and methods

This retrospective study includes patients with OF surgically treated at the Maxillofacial Traumatology Center of the “Magna Grecia” University Hospital of Catanzaro, from January 1st, 2007, to January 31st, 2021. The study was carried out according to the guidelines set out in the Helsinki Declaration and was approved by the Ethics Committee of the “Magna Grecia” University. Age, sex, fracture etiology, pattern and severity of the fracture, symptoms, comorbidity, timing of surgery, surgical approach, reconstruction materials, and short-term and long-term complications were collected. The inclusion and exclusion criteria of the study are summarized in Table 1.

**Table 1:** Inclusion and exclusion criteria of the study

Inclusion Criteria	Exclusion Criteria
Diagnosis of pure/ isolated orbital fractures.	History of surgery for orbital floor fractures.
Patients with complete clinical and/or radiological documentation.	Prior eye surgery.
12-month follow-up.	Non-cooperative patients.

All patients arrived, hemodynamically stable, from the Emergency Departments of other local hospitals with radiological exams and urgent specialist visits. After clinical and radiological consultation, patients were hospitalized in case of surgical indication. Before surgery, all patients were evaluated by a multi-specialist team (ophthalmologist, orthoptist, and neurosurgeon in case of neurocranial involvement). We also did the Hess-Lancaster screens and Hertel exophthalometry (in case of clinically clear enophthalmos). The instrumental examination of choice was thin-slice CT (1 mm) with acquisitions in axial, coronal, and sagittal and with 3D reconstructions. The severity of the fracture was assessed on the following radiological parameters: (1) herniation of the orbital fat into the maxillary sinus below; (2) herniation of the lower rectus muscle into the maxillary sinus below; (3) involvement of the infraorbital canal in the fracture rhyme. The main orbital floor fracture sites were classified into both sagittal and coronal planes in three portions: medial, lateral, or complete on the sagittal plane; anterior, posterior, or complete on the coronal plane. They were also classified according to the Harris classification.

All patients were treated under general anesthesia. We used two types of surgical approaches, sub-ciliary or infraorbital incision. Four different materials were used for the reconstruction of the orbital floor: (1) intraoperative bending of titanium mesh; (2) preformed titanium mesh modeled intraoperatively ; (3) absorbable membrane (Tutopatch®, the connective tissue of pure collagen preserved and dehydrated with organic solvents according to the patented process of preservation and sterilization Tutoplast®); (4) Patient-specific titanium mesh, modeled before surgery on a stereolithographic model obtained by 3D printing patient’s CT. Based on the time between trauma and surgery, patients were divided into two groups:

- Group A: early treatment, surgery performed within 2 weeks from the trauma.
- Group B: late treatment, surgery performed 2 weeks after trauma.

All patients underwent follow-ups for one year (Table 2).

**Table 2:** *Clinical-instrumental follow-up*

Time after surgery	Clinical-instrumental tests
*About 1 week after surgery*	*clinical check and removal of sutures (usually endodermal suture*
*After 15 days*	*clinical check*
*After 30 days*	*clinical check*
*After 3 months*	*clinical check and CT*
*After 6 months*	*clinical check, orthoptic evaluation, and execution of both Hess- Lancaster screens and Hertel exophthalometry (only in case of persistence of enophthalmos).*
*After 12 months*	*clinical check and CT*

At each clinical check, we evaluated the epicritic tactile sensitivity of the innervation region of the second branch of the trigeminal nerve (smear and/or pinch test and subjective evaluation questionnaire), extrinsic eye motility, the presence of diplopia and/or enophthalmos, and the presence of post-surgical complications. We evaluated post-surgical complications in the short term, within 15 days of surgery, and at a distance, more than 3 months after surgery. We also investigated the presence of infection, hematoma, palpebral retraction, ectropion, scars, and ptosis.

After 3 months and 1 year after surgery, a postoperative CT was obtained (in axial, coronal, and sagittal projections), and 3D reconstructions were made.

### Statistical analysis

We performed both descriptive and regressive statistical analyses on the recorded data.

We performed descriptive statistical analysis using central tendency indices (such as mean and range) and absolute and relative frequencies for categorical data. We performed regressive statistical analysis using the student’s t-test, calculated using the GraphPad program (GraphPad Company, San Diego, CA, USA).

## Results

From this retrospective analysis, it emerged that the two groups were fairly homogeneous according to the demographic characteristics of the patients treated surgically (age, sex, type of injury, etc.).

In the timeframe analyzed, we evaluated 1227 patients for orbital trauma and only 528 fulfilled the study’s inclusion criteria. The sample examined included 352 males (66.67%) and 176 females (33.33%), with an average age of 41.5 years and a range of 13–88 years. The most frequent cause of trauma was road accident (*n*.200, 37.88%), followed by domestic accidents (*n*.137, 25.95%), interpersonal violence (*n*.91, 17.23%), sports injuries (*n*.55, 10.42%) and work accidents (*n*.18, 3.41%). In a few cases, it was not possible to trace the etiology of the trauma (27 cases, 5.11%). Analyzing the correlation between gender and the mechanism of injury, road accidents were the most common cause of fracture in men (*n*.116, 32.95%), while domestic accidents were in women (*n*.80, 45.45%). Image 1 specifies the other mechanisms of injury based on gender.

**Fig. 1:**
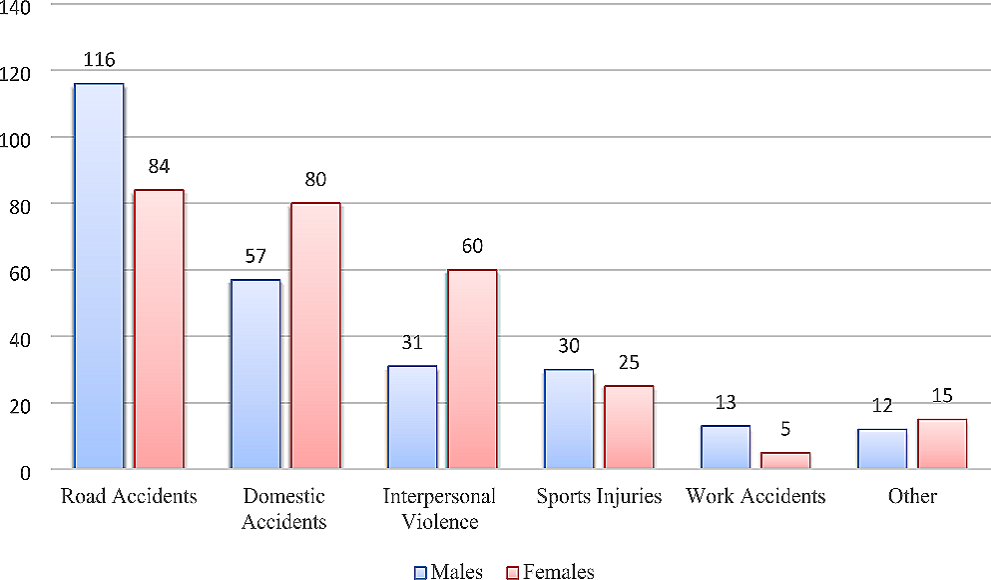
Mechanism of injury according to gender

In most cases, patients presented pure orbital fractures, in 401 cases (75.95%); only in 127 cases (24.05%) patients presented isolated orbital fractures. Of all pure orbital fractures (401 cases, 75.95%), 276 patients were affected by pure blow-out fractures (68.83%), 114 patients were affected by both blow-out and medial orbit wall fractures (28.68%), and 11 patients were affected by trap-door fractures (2.74%). Of all isolated orbital fractures (127 cases, 24.05%), 18 cases (13.79%) were associated with Orbital-Maxillo-Zygomatic (OMZ) complex fractures, and 11 cases (8.62%) were associated with the pan facial fracas. Data relating to the type of orbital fractures are summarized in Table 3.

**Table 3:** Orbital fracture pattern

Pure Orbital Fractures			Isolated orbital fractures	
Blow-out Fractures	Fractures of the floor and medial wall of the orbit	Trap-door Fractures	Orbital Fractures + OMZ complex fractures	Orbital Fractures in Pan Facial Fracas
276 patients (69.83%)	114 patients (28.68%)	11 patients (2.74%)	78 patients (61.42%)	49 patients (38.58%)
214 (%): Moderate grade according to Harris classification 176 (%): Severe grade according to Harris classification				
401 patients			**127 patients**	

The main signs and symptoms at diagnosis were periorbital edema (*n*.464, 87.88%), diplopia (*n*.382, 72.35%), enophthalmos (*n*.218, 41.29%), hypoaesthesia of the infraorbital nerve (*n*.282, 53.41%), extrinsic eye movement limitation (*n*.273, 51.70%). The presence of diplopia, enophthalmos, and/or restriction of extrinsic eye movement has determined the indication for surgical treatment. The presence of hypoesthesia alone, as an isolated symptom, has not indicated the surgery.

Considering the CT morphologic parameters, the anterior-medial portion of the orbital floor was the most affected, followed by the posterior-lateral portion.

On the coronal sections, the medial portion of the orbital floor was the most affected (144 cases, 52%), followed by the lateral portion (99 cases, 36%) and the entire floor (33 cases, 12%).

On the sagittal sections, the anterior portion of the orbital floor was the most affected (182 cases, 66%), followed by the rear portion (66 cases, 24%) and the entire floor (28 cases, 10%).

Considering the severity of the orbital floor fracture: 509 patients (96.55%) presented herniation of orbital fat into the below maxillary sinus, 273 patients (51.72%) presented herniation of the lower rectus muscle into the below maxillary sinus, 237 patients (44.83%) presented an involvement of the infraorbital canal by the fracture rhyme, and 18 patients (3.45%) presented a lower rectus muscle entrapment.

Regarding the surgical approach, the sub-ciliary incision is the most employed (419 cases, 79.36%), followed by the infraorbital one (109 cases, 20.64%). Thirty-six patients operated through sub-ciliary access (8.59%) have reported retraction of the lower eyelid, while none of the patients operated through infraorbital incision has gone through this type of complication. We reconstructed the orbital wall using different implants (Image 2), including intraoperative bending of titanium mesh (*n*.291 55.11%), preformed titanium mesh modeled intra-operatively (*n*.164, 31.06%), patient-specific titanium mesh (*n*.38, 7.20%), absorbable membrane type Tutopatch® (*n*.34, 6.63%).

**Fig. 2:**
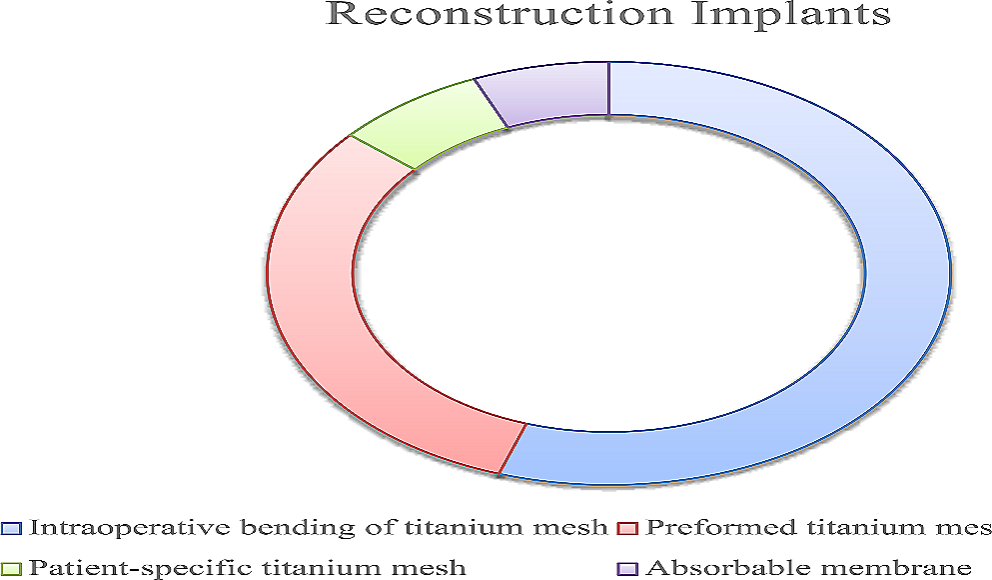
Different types of orbital floor reconstruction implants

Postoperative complications occurred mainly in isolated orbital fractures (where the orbit’s floor and medial wall are associated). In the first two weeks after surgery, we found a low degree of resolution of short-term diplopia, regardless of the severity of the fracture pattern. Two weeks after surgery, 124 patients with pure blow-out fractures (44.93%) had diplopia, and 39 patients with isolated orbital fractures (34.21%). Three months after surgery, diplopia was persistent in 52 patients with pure blow-out fractures (18.84%) and 24 patients with isolated orbital fractures (21.05%). This reversal has increased over time, in fact, 6 months after surgery, we recorded long-term postoperative diplopia in 36 patients with pure blow-out fractures (13.04%) and 18 patients with isolated orbital fractures (15.79%).

Data on pre-operative and post-operative diplopia are explained in Table 4.

**Table 4:** Pre- and post-operative diplopia depending on the type of blow-out fractures

	Preoperative entrapment of the lower rectus muscle	Preoperative Diplopia		Postoperative Diplopia: 2 weeks after surgery	Postoperative Diplopia: 3 months after surgery	Postoperative Diplopia: 6 months after surgery
*Pure Blow-out*	249	290	124		52	36
*Isolated orbital fractures*	24	92	39		24	18

We found a statistically significant correlation between the amount of lower rectus muscle herniation, visible at CT scans, and the presence of pre-operative (*p*-value = 0.00416) and postoperative (*p*-value = 0.00385) diplopia. Moreover, in the isolated orbital fractures, we observed a statistically significant correlation between the presence of short-term diplopia and its long-term persistence (*p*-value = 0.00513).

We recorded long-term postoperative enophthalmos in 9 patients (3.23%) with isolated orbital fractures and 18 (15.38%) with concurrent floor and medial wall orbit fractures.

Other long-term postoperative complications were less frequently detected: eyelid retraction (*n*.5; 8.62%), ectropion (*n*.2; 3.45%), lagophthalmos (*n*.2; 3.45%), ptosis (*n*.1; 1.72%), exophthalmos (*n*.1; 1.72%). Postoperative diplopia, restriction of extrinsic long-term eye movements, hypoesthesia, and enophthalmos were also evaluated about the timing of the surgery. These data are shown in Table 5.

**Table 5:** Post-operative complications of surgical timing

Timing of surgery	Group A: Patients treated within 15 days 409 (77.46%)	Group B: Patients treated after 15 days 119 (22.54%)
*Diplopia*	63 patients (15.40%)	27 patients (22.68%)
*Extrinsic eye movements limitation*	31 patients (7.58%)	16 patients (13.20%)
*Hypoesthesia*	99 patients (24.20%)	27 patients (22.69%)
*Enophthalmos*	54 patients (13.20%)	9 patients (7.56%)
***Total***	**247 patients (60.39%)**	**79 patients (66.39%)**

## Discussion

The incidence of OF is constantly increasing, with an incidence range of up to 70%, especially isolated orbital fractures /pure fractures. This is linked to the increase in road accidents, assaults, and domestic accidents; however, the mandatory use of appropriate personal protective equipment has proved effective in reducing the severity of facial injuries. The etiopathogenesis of such traumas is important for developing prevention strategies and descriptive studies are the first step towards improving treatment outcomes [12–16]. A reversal of the etiological trend occurred in our region during the 2020 lockdown, in which most traumas occurred in the domestic environment, in particular from interpersonal violence in women [17]; this inversion has also been reported in other Italian regions such as Campania and Umbria [18, 19]. Surgery in orbit fractures depends on clinical and radiological assessment. In the suspicion of OF the CT is the gold standard in terms of bone visualization, especially the coronal projection is the most effective to evaluate the orbital floor. The primary aim of surgery is to remedy functional and aesthetic damage. This is done by restoring normal orbital volume: repositioning soft tissues and freeing imprisoned muscles. However, surgery is not always indicated. In the present study, only patients with a medium to high grade according to the Harris classification [20] were treated: patients with moderate and/or severe orbital floor breakdown; patients with functional and/or sensorineural deficits (restriction of eye movements, diplopia, enophthalmos, infraorbital nerve hypoesthesia, etc.).

Examining the fracture pattern, pure blow-outs represent the predominant pattern, (401 cases): 69.83% of pure fractures and 52.27% of all fractures). When isolated orbital fractures are more often associated with OMZ complex fractures (14.77%). Regarding the fracture site, greater involvement of the anterior-medial portion was found (34.48%) which is associated with an increased risk of herniation of the orbital structures, especially the lower rectus muscle. The present study shows that there is statistical significance in the correlation between the severity of the lower rectus muscle herniation and the presence of pre-operative (*p*-value = 0.00416) and post-postoperative (*p*-value = 0.00385) diplopia. Thus, muscle herniation can be considered a negative prognostic factor for the recovery of diplopia. Our study supports the evidence of Ordon et al. [21] that the presence of diplopia by muscle herniation indicates surgery, regardless of the degree of herniation. The presence of edema and hematomas of peri-orbital tissues could mask or accentuate diplopia in case of minor breakdowns of OF.

We believe that early treatment leads to better outcomes in agreement with several authors who have recommended performing interventions within two weeks of the trauma [22]. Our indications for surgical treatment are persistent diplopia, increased orbital pressure, enophthalmos, visual deterioration, extraocular movement disorders, and hypoaesthesia of the infraorbital nerve, with the timing of repair of 5–7 seven days after injury.

To date, there is no consensus on repair time to achieve the best post-operative result. Several studies have shown that early intervention is associated with better results; however, other studies have suggested that subsequent surgery, after the disappearance of edema, can still produce even more satisfactory results. Although there is no precise date by which to perform surgery, the general recommendation is that surgery should be performed within a month to achieve clinically satisfactory results.

Regarding the surgical approach: we treated 419 patients with the sub-ciliary approach (79.36%) and 109 patients with the infraorbital incision (20.64%). The first caused more complications than the second. Thirty-six patients operated through sub-ciliary access (8.59%) reported the retraction of the lower eyelid while none of the patients operated through the infraorbital incision suffered this type of complication. The choice of reconstruction material is dictated by the type of fracture: we used non-resorbable material in the most severe cases; we used intraoperative bending of titanium mesh (291 patients, 55.11%), preformed titanium mesh (164 patients, 31.06%), and patient-specific titanium mesh shaped on 3D printed (38 patients, 7.20%).

The use of a patient-specific titanium mesh, modeled before surgery on a stereolithographic model obtained by 3D printing the patient’s CT scan, allowed for increased intra-operative efficiency and reduced post-operative complications. This remains to be confirmed by other future studies.

These materials allow for an optimal aesthetic and functional result if correctly positioned. By comparing the use of various non-absorbable materials, our study found no statistically significant difference in long-term complications, such as diplopia and enophthalmos. The same result was proposed by Strong et al. [23]. The long-term complications related to the timing of the surgery have shown different results depending on the symptom considered. Considering long-term post-operative enophthalmos (after 3 months from surgery), patients treated after 2 weeks showed a lower rate than those treated within two weeks (7,56% vs. 13,20%). This is related to the reabsorption of edema of periorbital soft tissues. Dal Canto et al., in a similar study, demonstrated how effective repair (with optimal functional and aesthetic recovery) can be achieved up to 29 days from trauma [24]. Other studies show how the early treatment of orbital fractures is associated with better results (both at functional and aesthetic levels) at a distance [13, 24–27]. Dal Canto et al. also highlight how a conservative approach in the first 15 days can help prevent unnecessary surgery when edema resolution does not show changes in visual function. Considering long-term post-operative diplopia and eye movements: patients treated two weeks after trauma have a higher rate of diplopia and limitation of eye movements than those treated within two weeks (respectively, diplopia 22.68% vs. 15.40%, and eye movements limitation 13.44% vs. 7.58%). These figures are consistent with the evidence of Prior et al. [28, 29]. Early surgical treatment reduces the likelihood of functional and structural damage to the lower rectus muscle [20, 24].

Fractures with orbital material herniation should be treated within 1–3 days after the trauma. Unfortunately, operating within this timeframe is not always logistically possible. However, you should not wait more than two weeks. In addition, in impure blow-out fractures, we observed a statistically significant correlation between the presence of short-term diplopia (immediately after surgery) and long-term diplopia (3 months after surgery), with a *p*-value of 0,00385. So, postoperative diplopia is a negative prognostic factor for long-term diplopia. Displaced fractures of the orbital walls when associated with other fractures of the maxillofacial area, require more complex surgical treatment and are often subject to the persistence of clinical-functional deficits in the long term (diplopia and enophthalmos).

## Conclusion

OF surgery involves the restoration of normal anatomy and orbital volume, avoiding aesthetic and functional damage, and is still much debated in the literature (approach, material used, and timing of repair). Our findings agree with other studies and provide important clinical information that will help future investigations of these lesions; they can be considered reliable since they come from one of the largest reference centers for maxillofacial trauma in Southern Italy.

Their epidemiology varies widely between populations due to socioeconomic differences, laws, and individual behavior. Although road safety legislation has proven to be effective in reducing the incidence of facial trauma, road accident trauma is still the most frequent cause and mainly involves men. Determining the specific etiopathogenesis of such traumas is important for devising prevention and treatment strategies. The proposed study highlighted the role of herniation of the lower rectus muscle as an important negative prognostic factor for long-term diplopia recovery. Long-term complications related to the timing of surgery have shown different results depending on the symptom considered: diplopia and limitation of eye movements have a higher rate in patients treated after two weeks of trauma; enophthalmos has a higher rate in patients treated within two weeks of trauma.

Open reduction and internal fixation are the treatments of choice for excellent effectiveness and low complication. However, surgery is not always indicated. Orbital floor exploration is indicated in patients with moderate and/or severe orbit floor damage, lower rectal entrapment, unresolvable diplopia, and significant enophthalmos. The use of patient-specific titanium mesh, modeled before surgery on a stereolithographic model obtained by 3D printing the patient’s CT, remains a fact to be confirmed by other future studies. Descriptive research like the one we propose is fundamental and the continuous sharing of data is certainly effective to define an optimal management path.

## Data Availability

The data presented in this study are available upon request from the corresponding author.
